# Long-term memory guidance of visuospatial attention in a change-detection paradigm

**DOI:** 10.3389/fpsyg.2014.00266

**Published:** 2014-04-01

**Authors:** Maya L. Rosen, Chantal E. Stern, David C. Somers

**Affiliations:** Department of Psychological and Brain Sciences, Boston University BostonMA, USA

**Keywords:** visual attention, change detection, memory cueing, visual working memory

## Abstract

Visual task performance is generally stronger in familiar environments. One reason for this familiarity benefit is that we learn where to direct our visual attention and effective attentional deployment enhances performance. Visual working memory plays a central role in supporting long-term memory guidance of visuospatial attention. We modified a change detection task to create a new paradigm for investigating long-term memory guidance of attention. During the training phase, subjects viewed images in a flicker paradigm and were asked to detect between one and three changes in the images. The test phase required subjects to detect a single change in a one-shot change detection task in which they held all possible locations of changes in visual working memory and deployed attention to those locations to determine if a change occurred. Subjects detected significantly more changes in images for which they had been trained to detect the changes, demonstrating that memory of the images guided subjects in deploying their attention. Moreover, capacity to detect changes was greater for images that had multiple changes during the training phase. In Experiment 2, we observed that capacity to detect changes for the 3-studied change condition increased significantly with more study exposures and capacity was significantly higher than 1, indicating that subjects were able to attend to more than one location. Together, these findings suggest memory and attentional systems interact via working memory such that long-term memory can be used to direct visual spatial attention to multiple locations based on previous experience.

## Introduction

In our everyday life, we are bombarded with visual information and we typically experience the world as if we have a complete picture. Yet, it is well documented that our visual working memory and attentional capacity are severely limited. Numerous experiments have observed an attentional capacity of approximately 4 ± 1 objects (e.g., Pylyshyn and Storm, 1988; Yantis, 1992; reviewed in Cowan, 2001). How can we reconcile our rich visual experience with the evidence of limited processing? While attention and short-term memory are limited resources, human long-term memory has a much higher capacity (e.g., Hollingworth, 2005; Brady et al., 2008). Therefore, it is reasonable to infer that humans exploit the massive capacity of long-term memory to aid visuospatial attention. Visual working memory is thought to act as the interface between long-term memory and attention such that items from long-term memory are called up into working memory and particular items can be held within the focus of attention as needed (Oberauer, 2002; Lewis-Peacock and Postle, 2012). Consider the experience of driving on an unfamiliar busy highway. It can be overwhelming. Even when road signs indicate that a lane is merging or an exit is approaching, it is easy to make mistakes due to the volume of new information. Once one has driven on this busy highway a few times, the task of maneuvering through the complex environment becomes much simpler. It is likely that part of this behavioral advantage is due to learning where to direct one's attention.

Long-term memory guided attention is an important and understudied form of visual working memory. A recent review (Hutchinson and Turk-Browne, 2012) has highlighted this issue and suggested that memory-guided attention should be added to the existing taxonomy of attention, which has historically solely focused on the division between exogenous, stimulus-guided attention and endogenous, goal-directed attention. There are many forms of memory-guided attention that have been studied in recent years. For instance, Soto et al. (2007) found that attention was drawn to a stimulus that was the same color as an item held in working memory. Subjects performed a target discrimination task within a working memory delayed match-to-sample paradigm for colored shapes. Reaction time was fastest when the target was embedded in the same colored shape that was held in working memory and slowest when the distractor was embedded in the colored shaped that was held in working memory. This finding provides evidence for the contents of working memory biasing attention. However, other studies (Downing and Dodds, 2004; Woodman and Luck, 2007) have found that when a distractor matches an item held in working memory, it speeds up reaction time. Woodman and Luck (2007) conclude that subjects are making voluntary shifts of attention away from a memory-matching stimulus based on the knowledge that the stimulus is a distractor. These findings suggest that holding information in working memory is not in and of itself sufficient to guide attention (Woodman and Luck, 2007; Olivers, 2009). It has been argued that after only moderate amounts of exposure, long-term memory takes over for working-memory in guiding attention (Woodman et al., 2013). Here, we further argue that long-term memory can bias attention by bringing relevant items into visual working memory.

Several studies have shown a behavioral advantage of attention when a subject has previous experience with a stimulus. Most notably, Chun and Jiang (1998) found in a visual search paradigm that the latency to detect a target decreases when targets appear in consistent spatial locations, even when subjects are unaware of this consistency. This effect, which they named “contextual cueing,” provides evidence that the human brain implicitly uses prior experience to direct attention (Brockmole and Henderson, 2006; Chun and Turk-Browne, 2007). Furthermore, Werner and Thies (2000) used a change-detection flicker paradigm in football experts and novices and found that change detection in novel football scenes was more rapid in experts than novices. This finding provides evidence that humans can also generalize knowledge from previous experience to direct spatial attention.

Another mechanism that supports processing in complex visual environments is divided spatial attention. Numerous lines of evidence indicate that humans and non-human primates are able to divide visual attention into multiple discrete spotlights (Shaw and Shaw, 1977; Awh and Pashler, 2000; Müller et al., 2003; McMains and Somers, 2004; Cavanagh and Alvarez, 2005; McMains and Somers, 2005; Alvarez and Franconeri, 2007; Adamo et al., 2008; Cave et al., 2010; Niebergall et al., 2011). Although some have questioned whether multiple object selection reflects parallel or very rapid serial processing (e.g., Tsal, 1983; Jans et al., 2010), there is clear evidence that such selection can provide behavioral advantages in the presence of many distracting stimuli (Awh and Pashler, 2000; Cavanagh and Alvarez, 2005; McMains and Somers, 2005; Bettencourt and Somers, 2009). Prior studies have investigated multifocal attention using explicit cues either at the locations of interest (exogenous) or at central location (endogenous), but none have explicitly investigated long-term memory-driven orienting of spatial attention.

It has been proposed that working memory, the ability to hold information in mind and manipulate it in some way, is an emergent property of interactions between attention and long-term memory (Oberauer, 2002) and that working memory consists of two components, those items that are in the focus of attention which has a limited capacity, and those items that are outside the focus of attention, but in an active state of long-term memory and thus more easily accessible (Cowan, 1988). It has been debated whether more than one object can simultaneously be within the focus of attention (Oberauer and Bialkova, 2009; Gilchrist and Cowan, 2011). In a recent review of the literature, Oberauer (2013) reanalyzed data from several studies to try to determine whether more than one item can be held simultaneously in the focus of attention. He concluded that humans are able to simultaneously attend to multiple distinct objects in working memory.

Here, we developed a novel paradigm to investigate long-term memory guidance of visual spatial attention. Our goals were to tightly control the time window in which attention must be deployed and to investigate the deployment of LTM-guided attention to multiple locations. To this end, we adapted a popular change detection/change blindness paradigm. Change blindness, the tendency of subjects not to detect differences between stimuli, occurs even when subjects are actively searching for a change (Levin and Simons, 1997; Rensink et al., 1997; Simons and Levin, 1998; Simons, 2000). However, when participants find a change, it becomes very obvious to them. To exploit this phenomenon, we designed a paradigm in which participants studied the location of changes in complex outdoor scenes in a standard change detection flicker paradigm (Rensink et al., 1997). Then, subjects were tested in a one-shot change detection task on the images that they studied previously. In this modified change-detection paradigm, subjects view a scene and must retrieve from long-term memory the location(s) of the potential change(s), they must then hold those location(s) in visual working memory until the image disappears and the probe image appears and they may compare the location(s) in the probe image to what is being held in working memory. Additionally, the brief target scene presentation of this paradigm permits investigation of the ability to simultaneously deploy attention to multiple discrete remembered locations in complex real-world scenes.

## Experiment 1

In the first experiment, we presented subjects with images of scenes in a change detection task. We manipulated the number of studied changes in each scene (0, 1, 2, or 3). At test, subjects were required to covertly attend to the remembered location(s) in order to determine capacity in this task. The experiment consisted of a study phase, in which subjects viewed the image changes repeating in a flicker-paradigm loop, and a one-shot test phase, in which subjects had to detect a change that occurred on 50% of trials. We hypothesized that studying changes would increase subjects' sensitivity to detect changes. Additionally, we hypothesized that subjects' capacity would be higher in the multiple studied change conditions (2- and 3-studied changes) than in the single studied change condition.

### Materials and methods

#### Subjects

Subjects were recruited from the Boston University community and received course credit or $10 compensation for their participation. This research was approved by Boston University Charles River Campus Institutional Review Board and all subjects gave written informed consent. Thirty healthy subjects participated in Experiment 1 (mean age 18.8 years, 8 males). The data from 6 subjects (2 males) were excluded from analyses because subjects failed to hold fixation during the testing period.

#### Study period

**Figure 1:**
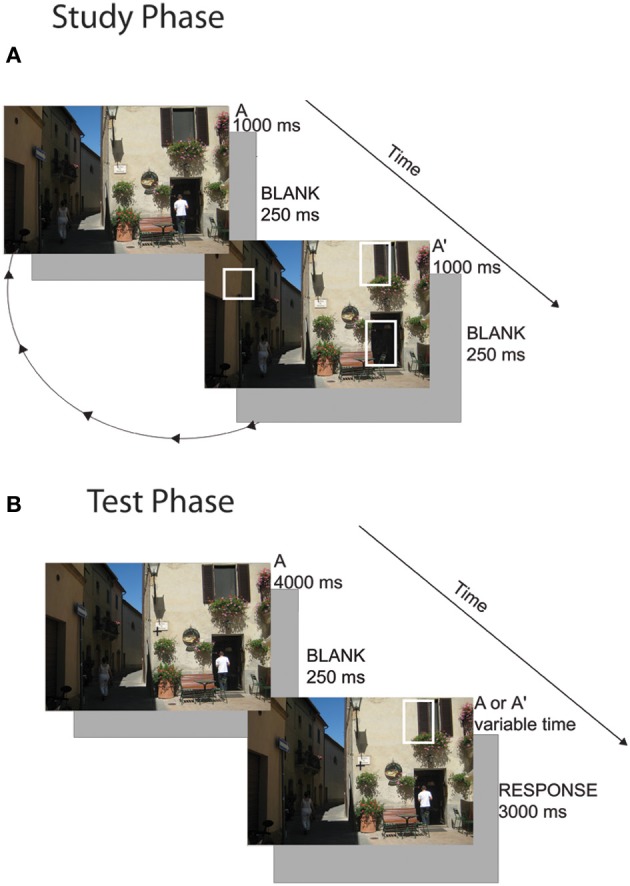
**Change detection task. (A)** Cycling Study Phase: during the study phase, subjects freely viewed images in a flicker paradigm. They were instructed to click on the changes as they found them. In Experiment 1, subjects viewed 80 scenes with 0, 1, 2, or 3 changes. In Experiment 2, subjects viewed 20 scenes with 3 changes and studied the set three times. **(B)** One-shot Test Phase: subjects held central fixation while a novel or familiar scene image appeared. They were instructed to covertly attend to where they thought a change might occur. The image disappeared and then flashed briefly with 0 or 1 change and then disappeared again. The subject had to determine whether a change occurred.

Subjects freely viewed scenes in the change detection flicker paradigm. Scenes had been used in a previous experiment (Schon et al., 2004) and were edited in Adobe Photoshop to create multiple versions of the scene with spatially discrete changes (e.g., a car changing color, a building disappearing). Subjects viewed 80 scenes in total with 0, 1, 2, or 3 changes (20 per condition). Twenty unique scenes were used in each condition and were not counterbalanced across subjects (i.e., all subjects viewed the same 20 scenes as all other subjects in the 3-change condition). This is a limitation of the study design because the differences seen between the responses to each condition could be due to differences in the scenes themselves. However, we note that in many cases, the images with more scene changes (2- and 3-studied change images) had subtler and smaller changes than in the 1- and 0-studied change conditions. This may have in fact reduced our ability to find differences in capacity between conditions rather than falsely inflating the differences. Note that subjects were given equal exposure to scenes with and without changes. Subjects were instructed to visually search the image until they found the change(s). Once they detected a change, they were instructed to click on it with the mouse. If they determined that no change occurred between the scenes they were instructed to click anywhere outside of the image. Trials were presented in blocks of 0- and 1-change images and blocks of 2- and 3-change images. A cue appeared at the beginning of the block that indicated how many changes the subject should be looking for (i.e., “0 or 1 changes” or “2 or 3 changes”). Subjects were also informed that the goal was to learn the changes because they would be tested on them. On a given trial, a picture of a scene (Scene A) flashed on the screen for 1000 ms, then a blank screen appeared for 250 ms and then a potentially altered scene (Scene A') appeared for 1000 ms, and was then replaced with a blank screen for another 250 ms. This cycle continued for 16 s after which a 10 s reveal period occurred. During the reveal period, Scene A appeared for 1000 ms followed immediately by Scene A' for 1000 ms and so on. Because no blank screen occurred between the presentation of Scene A and Scene A', any changes that were present became very apparent. Subjects were instructed to click on the changes at any point during the 26-s presentation (during the initial flicker period or the reveal period) (Figure 1A). Images subtended approximately 12° × 8° of visual angle. Visual stimuli were presented on a Mac Pro using the Vision Egg software package (Straw, 2008).

#### Test period

A given image appeared on screen for 4000 ms. Subjects were instructed to use this time to covertly direct their attention to all possible location(s) of the changes, in preparation for a one-shot change detection. Eye position was manually monitored by the experimenter throughout the task using an eye camera to ensure that subjects maintained fixation throughout the test period. Subjects were excluded if they did not hold adequate fixation for at least 95% of trials. If they had not previously studied a change in that scene (0-change condition), they were instructed to attempt to diffusely direct their attention to the entire scene. The image then disappeared for 250 ms and appeared (possibly changed) for 500 ms. There was a 50% chance that a single change occurred and a 50% chance of no change occurring in the second image presentation. For images where subjects studied 1, 2, or 3 changes, if a change occurred, it was always a studied change. In the images that subjects saw no changes during the study period, test phase changes were always unstudied changes. Subjects were given 3000 ms to make a two-alternative forced choice whether or not a change occurred (Figure 1B).

Sensitivity to detect changes was calculated using *d′*:

(1) $$d^{\prime }=z(HR)-z(FAR)$$

where *z*(*HR*) and *z*(*FAR*) are the inverse of the cumulative Gaussian distribution of the hit rate and false alarm rate, respectively.

In order to evaluate whether subjects were able to hold more than one location in visual working memory in the 2- and 3-studied change conditions, it is critical to take into account set size when assessing performance on task that requires divided attention. Cowan's *k* is a well-established method for estimating capacity in change-detection paradigms (Pashler, 1988; Cowan, 2001; Todd and Marois, 2004; Xu and Chun, 2006). *k* was calculated:

(2) k=(HR−FAR)×SS,

where *HR* is the hit rate, *FAR* is the false alarm rate and *SS* is the set size. *k* provides an estimate of the number of locations to which the subject is effectively holding in the focus of attention.

### Results: experiment 1

Although subjects had equal exposure to all images, exposure to image changes during the study phase significantly improved their change detection performance during the test phase. The mean sensitivity (*d′*) to detect the changes for the 0-, 1-, 2-, and 3-studied change conditions was 0.60 ± 0.12, 1.89 ± 0.15, 1.47 ± 0.17, and 0.89 ± 0.15, respectively. A one-sample *t*-test revealed that subjects performed this task significantly better than chance (*d′* = 0) in the 0-studied change condition [*t*_(23)_ = 4.78, *p* = 0.0002]. Exposure to changes in images prior to the test period significantly modulated subsequent sensitivity to detect these changes. The sensitivity to detect changes in each of the (1-,2-,3-) studied change conditions was significantly higher than the 0-studied condition [*t*_(23)_ = 8.14, *p* < 0.0001, *t*_(23)_ = 5.43, *p* < 0.0001, *t*_(23)_ = 2.16, *p* = 0.041, for 1-, 2-, and 3-changes respectively, all *p*-values are Holm-Bonferroni corrected for three one-sample comparisons]. These results indicate that prior exposure to the location of changes helps to support visual working memory to guide visuospatial attention. Subjects are able to rapidly remember the studied location(s) and deploy their attention to those locations to monitor whether a change occurred. It should be noted that as the number of locations to which the subject needed to attend increased, the sensitivity to detect those changes decreased. The *d′* for the 1-studied change condition was significantly higher than for the 2- and 3-studied change conditions [*t*_(23)_ = 2.25, *p* = 0.034, *t*_(23)_ = 7.27, *p* < 0.0001, respectively] and the *d′* for the 2-studied change condition was significantly higher than the *d′* for the 3-studied change condition [*t*_(23)_ = 2.73, *p* = 0.024, all *p*-values are Holm-Bonferroni corrected for 3 between-condition comparisons]. Clearly, as subjects had more trained locations to which to direct their attention, sensitivity declined. The hit rate was significantly higher for all studied change conditions (1-, 2-, and 3-change conditions) compared to the no-studied change condition [Mean(1-change) = 0.77 ± 0.03, Mean(2-change) = 0.69 ± 0.04, Mean(3-change) = 0.55 ± 0.07, compared to Mean(0-studied change) = 0.24 ± 0.04, all *p* < 0.05, Holm-Bonferroni corrected]. Additionally, the false alarm rate was also significantly higher in all the studied change conditions compared to the 0-studied change condition [Mean(1-change) = 0.17 ± 0.032, Mean(2-change) = 0.22 ± 0.04, Mean(3-change) = 0.27 ± 0.07, compared to Mean (0-studied change) = 0.09 ± 0.01, all *p* < 0.05, Holm-Bonferroni corrected].

**Figure 2:**
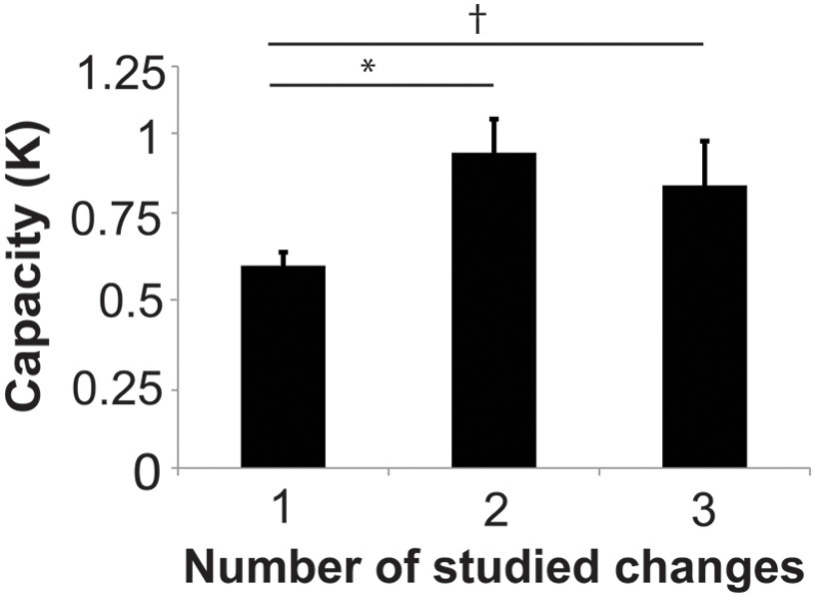
**Capacity (K) for remembered changes in studied images (1, 2, or 3-change conditions)**. ^*^Indicates *p* < 0.05, Holm-Bonferroni corrected. ^†^Indicates *p* < 0.1.

In order to evaluate whether subjects were able to hold more than one location in visual working memory in the 2- and 3-studied change conditions, it is critical to take into account set size when assessing performance on task that requires divided attention. Cowan's *k* is a well-established method for estimating capacity in change-detection paradigms (Pashler, 1988; Cowan, 2001; Todd and Marois, 2004; Xu and Chun, 2006). *k* for each condition was 0.16 ± 0.03, 0.60 ± 0.04, 0.94 ± 0.10, and 0.84 ± 0.13 for the 0-, 1-, 2-, and 3-studied change conditions, respectively. *k* in 2-studied change condition was significantly higher than the 1-studied change condition [*t*_(23)_ = 3.45, *p* = 0.0066, Holm-Bonferroni corrected] and there was a trend toward a higher k-score in the 3-change condition compared to the 1-change condition [*t*_(23)_ = 2.09, *p* = 0.095, corrected]. There was no significant difference in *k* for the 2-studied change condition compared to the 3-studied change condition [*t*_(23)_ = 0.617, *p* = 0.543, corrected] (Figure 2).

These findings suggest that subjects were able to attend to more locations when they studied 2 or 3 changes compared to when they studied 1 location. However, since k did not exceed 1 in any condition, this leaves doubt about the ability to simultaneously attend to multiple target locations in this paradigm. One possible explanation for the low capacity in Experiment 1 is that subjects did not remember the location of the changes in some images. Given the large number of images studied (80), it is likely that one exposure during the study phase is not adequate to strongly encode multiple image change locations. A second concern is that the 500 ms probe image presentation time might be sufficiently long to permit subjects to rapidly switch the focus of attention from one spatial location to another (e.g., Tsal, 1983; Sperling and Weichselgartner, 1995). We address these issues in Experiment 2 by increasing the number of study exposures for each image during the training phase and by parametrically varying the probe presentation time during the test phase.

## Experiment 2

In Experiment 2, subjects studied each scene three times. We measured latency to find each change during the study periods. We used these latencies to determine whether subjects successfully learned the locations of each change. Subjects then underwent two test phases with different probe presentation times (150, 250, and 500 ms). We expect that subjects will learn more change locations with each viewing of the scenes. Furthermore, we hypothesized that the increased exposure to the images will result in increased capacity compared to that of the subjects' in Experiment 1. Finally, we explored whether subjects exhibit similarly high capacities with shorter probe presentation times (150 and 250 ms probes) compared to the longer probe presentation times (500 ms probe). The motivation for varying the probe presentation time was to investigate possible effects of a rapidly moving attentional spotlight (e.g., Tsal, 1983). Volitional moves of attention are generally believed to take a relatively fixed amount of time, regardless of the distance between the attended and to-be attended locations (Yantis, 1988; Sperling and Weichselgartner, 1995; Cave and Bichot, 1999), and thus suggests that the attention does not need to pass through the space between an attended location and a to-be attended location. Rather, attentional shifts can be made in a quantal fashion. These volitional shifts are generally believed to take approximately 200 ms (Wolfe et al., 2000).

### Materials and methods: experiment 2

#### Subjects

Sixty-two healthy subjects (mean age 22.8 years, 22 males) participated in Experiment 2. Data from 15 subjects (4 males) were excluded due to inadequate fixation during the test period.

#### Study period

Subjects viewed 20 scenes using the same flicker paradigm described in Experiment 1. During the study phase, all 20 scenes contained three changes. Reaction time to find and click on the location of each of the 3 changes was recorded. Subjects viewed all 20 scenes in the flicker paradigm three times to ensure that they learned each of the locations of the changes.

In order to estimate the number of changes remembered after each exposure, we created a simple linear model which assumes that the average reaction times reflect a weighted average of the time to respond to a remembered change and the time to search for, find and respond to an unremembered change:

(3) $$Avg\Delta RT(x)=P_{M}(x)\times RT_{M}+(1-P_{M}(x))\times RT_{S},$$

where, *P*_*M*_ is the proportion of changes remembered. *RT*_*M*_ is the reaction time for remembered changes, which we assumed to be the group average fastest recorded reaction time (difference between detecting one change and detecting the next change, 1.65 s). *RT*_*S*_ is the reaction time for visual search trials, which we assume to be the average difference in reaction times for all detected changes during the first exposure to the images (i.e., the average difference in RT between locating the 1st and 2nd change and the *RT* difference between locating the 2nd and 3rd change, 6.52 s). The 1st, 2nd, and 3rd changes were defined simply by the order in which subjects clicked on each change, not predefined by the experimenter. On the first exposure the subjects cannot be using memory and thus must be searching. We can rearrange the terms of this equation to estimate the proportion of locations remembered after each exposure, *P*_*M*_(*x*):

(4) $$P_{M}(x)=\frac{RT_{S}-Avg\Delta RT(x)}{RT_{S}-RT_{M}},$$

*P*_*M*_ was calculated separately for each of the 1st, 2nd, and 3rd changes. We can then estimate how many changes each subject remembers at each exposure by adding the proportion of trials in which subjects are relying on their memory for the 1st, 2nd, and 3rd change using the following equation:

(5) $$C=P_{M}(1^{st})+P_{M}(2^{nd})+P_{M}(3^{rd}),$$

where *C* is the estimated number of learned changes.

#### Test period

The test period paradigm was similar to that of Experiment 1 with two major differences. As in the first experiment, the original scene was presented for 4000 ms. After a 250 ms blank screen, the second image was presented for one of three pseudo randomly chosen durations (150, 250, or 500 ms). Additionally in Experiment 2, subjects were given one practice test, followed by two test phases with all 20 images appearing twice and the probe durations intermixed. We collapse results of the two test phases because performance did not differ on these two tests.

### Results and discussion: experiment 2

**Figure 3:**
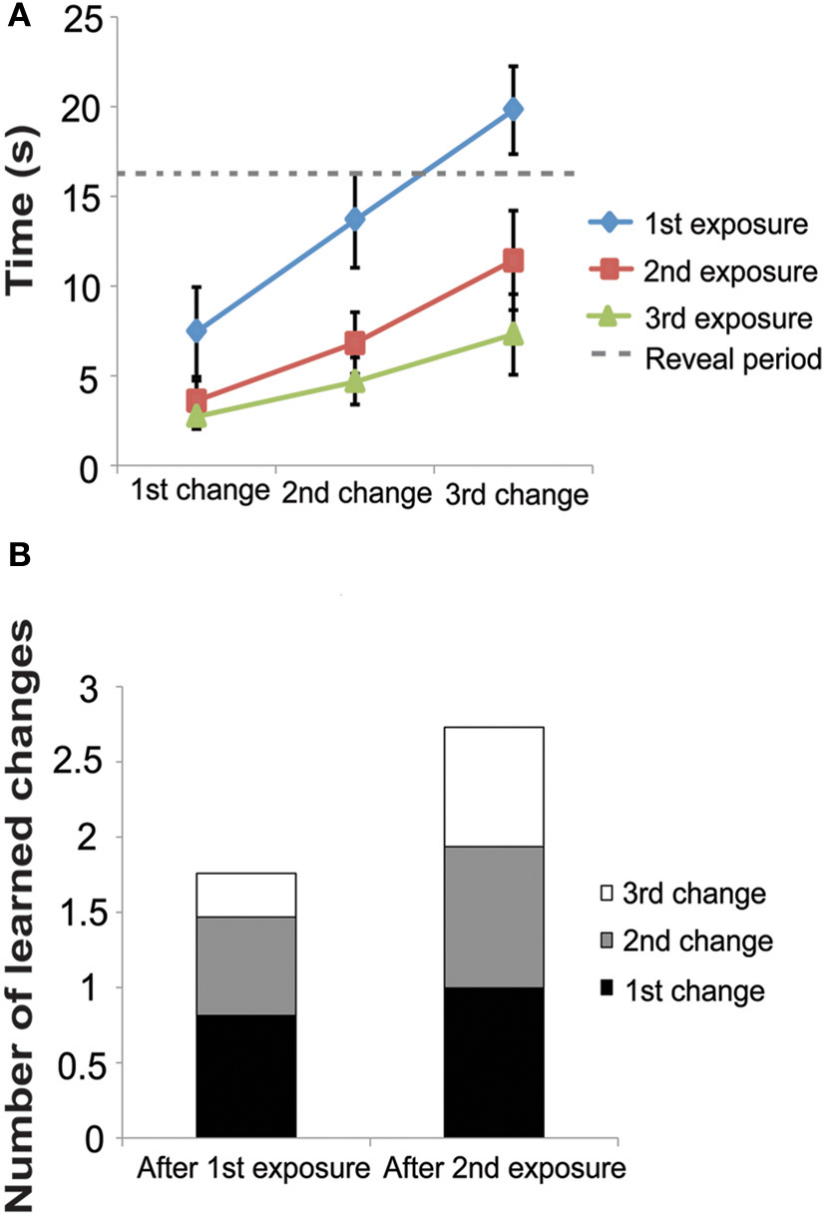
**Experiment 2 Study Phase. (A)** Latency to find changes decreased with each exposure in the study phase. Note that all three changes were presented simultaneously (see Figure 1A) and thus subjects responded to each change as they noticed it. **(B)** Using our model, Equation (4), we estimate that after the second exposure subjects remember almost all three changes. These data are taken from the time to detect the each change after each exposure. The first bar labeled “After 1st Exposure” was taken from latency to find each change during the second exposure and the second bar labeled “After 2nd Exposure” was taken from the latency to find each change during the third exposure. Subjects were not exposed to the images a fourth time and therefore we do not have the latency to estimate each change after the 3rd exposure.

Study phase: with each study exposure, latency to find the changes decreased (Figure 3A). On the first exposure, the latency to find the 1st, 2nd, and 3rd changes were 7.00 ± 0.42 s, 13.07 ± 0.46 s, 19.57 ± 0.42 s, respectively. On the second exposure, latency to find the changes was faster (3.44 ± 0.19 s, 6.59 ± 0.36 s, and 11.35 ± 0.55 s for the 1st, 2nd, and 3rd changes, respectively.) In the third and final study phase, latency was even faster (2.64 ± 0.12 s, 4.55 ± 0.25 s, and 7.08 ± 0.44 s, for the 1st, 2nd, and 3rd changes, respectively). During the first exposure, subjects found some of the changes after the reveal period, but during the second and third exposures, subjects were able to find all changes before the reveal period.

Using the above formula to estimate *C* (the number of learned changes), we estimate that after the 1st exposure, subjects had learned approximately 1.9 changes and after the second exposure, subjects had learned approximately 2.8 changes (Figure 3B). These data demonstrate that subjects did not remember the location and identity of all 3 changes after just one exposure. This fact likely contributed to the low k-score found in the 3-change condition in Experiment 1.

#### Test period

**Figure 4:**
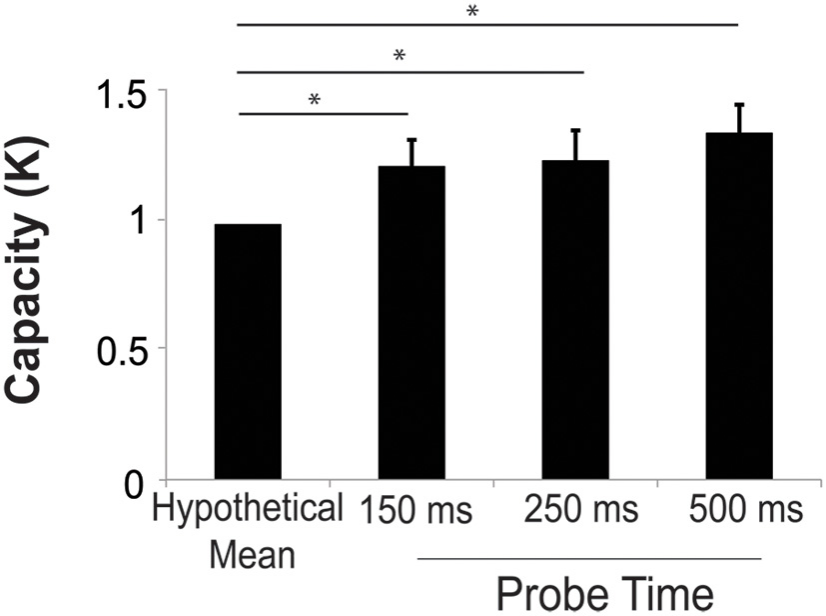
**Capacity increased significantly above a hypothetical mean of 1 at all probe durations, suggesting that subjects could successfully deploy their attention to more than one remembered location**. Error bars are *s.e.m*. ^*^p < 0.05.

*K*-score was calculated at each probe presentation time. The mean *k* was 1.36 ± 0.11, 1.25 ± 0.12, and 1.23 ± 0.11 for the 500, 250, and 150 ms probe conditions, respectively. We performed a two-sample *t*-test comparing the k-score for the 500 ms probe in Experiment 2 (20 scenes each with 3 changes) to the 3-change condition (20 scenes) in Experiment 1. The *k* in Experiment 2 was significantly higher than that of Experiment 1 [*t*_(69)_ = 2.84, *p* = 0.0076]. This confirms the finding from model analysis of the study phase that increased study exposures improved capacity. We performed a one-sample *t*-test to compare the average *k* in the test period in Experiment 2 to hypothetical mean of 1 in order to determine whether subjects could effectively deploy their attention to more than one location. At all three probe durations, the k-score was significantly higher than 1 [*t*_(46)_ = 3.10, *t*_(46)_ = 2.10, *t*_(46)_ = 2.25, all *p* < 0.05 for the 500, 250, and 150 ms probe durations respectively]. There were no significant differences in *k* at the three different probe presentation durations (Figure 4). The mean hit rates were 0.66 ± 0.3, 0.62 ± 0.3, and 0.56 ± 0.3 for the 500, 250, and 150 ms probe durations. The mean false alarm rates were 0.21 ± 0.03, 0.21 ± 0.03, and 0.15 ± 0.03 for the 500, 250, and 150 ms probe durations, respectively.

These findings indicate that subjects were able to successfully detect more than one change at all probe durations. However, the number of successfully detected changes in this paradigm (Figure 4) is lower than the number of change locations that subjects learned in the study phase (Figure 3B). In the study phase analysis we estimated that subjects required 1.65 s per change location to identify changes, while in the test phase, subjects had only 150–500 ms to identify changes. Moreover, subjects freely viewed images in the study phase, but were required to maintain central fixation and detect peripheral changes in the test phase. Given these large temporal and spatial advantages, it is not surprising that performance in the study phase exceeded that in the test phase.

There has been a long debate in the literature about the speed at which attention may move (e.g., Reeves and Sperling, 1986; Sperling and Weichselgartner, 1995; Cave and Bichot, 1999; Wolfe et al., 2000; Cave et al., 2010; Jans et al., 2010). Volitional moves of attention are widely accepted to take 200 ms or longer (Wolfe et al., 2000; Cave et al., 2010), regardless of distance between the locations. We note that we failed to observe any significant difference in capacity as we varied probe time between 150 and 500 ms and thus our results across all probe times are not consistent with a rapidly moving spotlight interpretation for this paradigm. Rather, these results provide evidence that subjects can divide attention based on a memory associated with a particular image. However, we note that this finding is a null result and therefore should be interpreted with caution.

## General discussion

In a series of two experiments, we used a modified change detection paradigm to explore how memory helps to guide spatial attention. In Experiment 1, participants were trained on a change-detection flicker paradigm with complex visual scenes that contained 0, 1, 2, or 3 changes at different spatial locations. During the test phase, a previously studied scene was presented statically and subjects were instructed to covertly direct their attention to all the possible locations in which a change had occurred during the study phase. The scene would disappear and then reappear after a short blank period, and participants responded with a two-alternative forced choice whether or not they detected a change (Change, No change). Results indicate that the number of successfully attended items significantly increased in the multiple-change conditions (2- or 3-studied changes) compared with the single change condition. In Experiment 2, we increased the number of study exposures and found that subjects were faster at finding the changes with every study exposure. Change detection performance increased to significantly above 1 with increased study exposures. These findings suggest that humans can covertly attend to more than one remembered location. Moreover, this work introduces a new paradigm for investigating interactions between long-term memory for visual scenes and visual working memory deployment of visuospatial attention.

Our findings also provide additional evidence that humans can simultaneously hold more than one location in the focus of attention. The results indicate that in order to successfully divide attention in this task, the memories must be sufficiently robust, which was accomplished by exposing subjects to the changes multiple times. In contrast, a rapidly shifting attentional spotlight model would predict more items would be attended with the longer probe duration; however, we failed to observe an effect of probe duration in the range of 150–500 ms in Experiment 2. This indicates that rapid shifts of attention likely did not play a role in our results. We note that this finding is a null result and should be interpreted with caution. Only in the free-viewing conditions of the long-duration study phase trials did we observe an effect of probe duration; it is not surprising that the opportunity to move ones eyes to a potential target location enhanced performance. However, in the critical test phase, eye movement controls insured that subjects did not move their eyes; under these conditions, we observed no evidence for rapid shifts of covert attention. Our results also indicate that even inexperienced observers can divide spatial attention based on memory cues. It is possible that minimal training using another covert attention task would further improve subjects' ability to perform this task. Another possibility is that a single focus of attention is more broadly distributed in the multiple target conditions, selecting targets and intervening distractors. These data cannot rule out this possibility and future studies should attempt to further tease apart this idea.

The contextual cueing paradigm (e.g., Chun and Jiang, 1998) has provided firm evidence that humans can use a familiar context to direct their attention to a particular spatial location. More recently, Conci and Müller (2012) showed that subjects could be contextually cued to multiple locations in the same context. This recent paper provides additional support for our finding that subjects can rapidly update attention to multiple locations based on a particular remembered stimulus. Conci and Müller used standard contextual cueing stimuli wherein subjects had to find a target letter (T) among several distractor letters (L). They found a reliable contextual cueing effect to multiple locations using these stimuli. Brockmole and Henderson (2006) showed the contextual cueing effect holds in real-world scenes. The present study demonstrates that humans are able to simultaneously attend to more than one remembered location using visually complex, real-world scenes. Contextual cueing effects are often attributed to implicit memory mechanisms (Chun and Jiang, 1998). Here, subjects report explicitly remembering the location of the change(s) and directing their attention based on that explicit memory. While the current study did not contain a test of explicit memory and our assumption that subjects were using explicit memory to guide their attention is based on anecdotal evidence, the type of stimuli used (complex visual scenes), the number of exposures, and the depth of encoding required by repeated visual search, encourage the formation of explicit memories. Unlike in the visual search paradigm of contextual cueing, in our paradigm subjects have only one chance to detect a change in this task and therefore must effectively deploy their attention to the remembered locations rapidly. Therefore, our paradigm provides a unique method by which to probe questions regarding guidance of spatial attention by using explicit, declarative memory.

It is also noteworthy that we adapted the flicker paradigm (Rensink et al., 1997) to develop a new paradigm that provides tight control over the duration of attentional selection. The short exposure of the (potentially changed) probe stimulus limits the movement of attention. A one-shot change detection paradigm also has been used in visual short-term memory (VSTM) paradigms (e.g., Luck and Vogel, 1997). Many studies of visual working memory have focused solely on the contributions of short-term memory. Here, we explicitly investigate the interactions between long-term memory, working memory and attention. Subjects must retrieve the location(s) of the change(s) from long-term memory and hold them in the focus of attention in visual working memory until the probe image appears and they can make a decision about whether a change occurred. Notably, the tight timing control afforded by this paradigm may prove useful in fMRI experiments, a methodology that has only coarse temporal control.

In a prior study of contextual cueing using functional MRI, Summerfield et al. (2006) found that memory-guided attention recruits largely the same brain networks—notably the intraparietal sulcus and frontal eye fields—recruited by visually guided attention; however, memory-guided attention also recruited the left hippocampus while visually-guided attention did not. Orbitofrontal cortex has also been implicated along with the hippocampus in context-dependent retrieval tasks (Ross et al., 2011; Brown et al., 2012). The present study lays the foundation for future investigations of the neural mechanisms by which memory guides spatial attention.

### Conflict of interest statement

The authors declare that the research was conducted in the absence of any commercial or financial relationships that could be construed as a potential conflict of interest.
